# Seasonal and spatial variation of surface current in the Pemba Channel, Tanzania

**DOI:** 10.1371/journal.pone.0210303

**Published:** 2019-01-07

**Authors:** Masumbuko Semba, Rick Lumpkin, Ismael Kimirei, Yohanna Shaghude, Ntahondi Nyandwi

**Affiliations:** 1 Institute of Marine Sciences, Zanzibar, Tanzania; 2 Nelson Mandela African Institution of Science and Technology, Arusha, Tanzania; 3 NOAA/Atlantic Oceanographic and Meteorological Laboratory, Miami, FL, United States of America; 4 Tanzania Fisheries Research Institute (TAFIRI), Kigoma, Tanzania; University of Vigo, SPAIN

## Abstract

The surface current speeds within the Pemba channel were examined using 24 years of drifter data received from the Global Drifter Program. This study aimed to uncover varying surface current in the Pemba Channel in different seasons. The results revealed the Pemba Channel experiences relatively higher median surface current speeds during the southeast (SE) monsoon season compared to the northeast (NE) and inter-monsoon (IN) periods. The strongest current speeds were confined in waters deeper than 200 meters between ~39.4°E and 39.7°E. These results prove that surface currents from the drifters can be used to uncover the patterns of surface circulation even in areas where in-situ measurements are scarce.

## Introduction

Surface currents of the ocean are moving parcels of water under the influence of wind, tides, earth’s rotation (Coriolis Effect) and difference in water density from one location to another [1]. The bottom topography and shape of the ocean basins and nearby landmasses also influence the ocean currents [2]. These forces and their physical characteristics affect the size, shape, speed and direction of ocean currents both in time and space. While the physical features govern the way surface currents vary in space [3], the general weather pattern influences the way surface currents vary in time and space [4].

Currents are important in coastal and marine ecosystems as they transport water masses and redistribute heat, nutrients, salinity and oxygen in the ocean [5]. Ocean currents are also critical in the dispersal and transport of larval forms of many marine living organisms [6]. They also distribute nutrients needed for the growth of phytoplankton, thus strengthening food production at different marine trophic levels [7]. Various socio-economic activities depend on a clear understanding of surface currents. These include shipping, rescue operations, transport and forecast dispersing of floating materials like plastic or oil [8]. Thus, understanding the ocean current circulation patterns is vital for the marine spatial planning, proper management of coastal and marine resources and the environment.

However, information of clear pathways of ocean currents along the coastal waters of Tanzania is limited. Several previous studies used a regional ocean modeling tool to study the seasonal changes in water circulation, temperature and salinity along the coastal waters of Tanzania east of the Zanzibar islands [9]. Other studies have used Acoustic Doppler Current Profiler (ADCP) data to explore the vertical flow structures of the East African Coastal Current (EACC) over the monsoon seasons along the Zanzibar Channel [4]. Moreover, other studies integrated ocean models and in-situ measurements to study the circulation dynamics along the coast of Tanzania [10]. In their study, [10] examined seasonal and tidal changes of the Zanzibar channel using the Regional Ocean modeling tool and noted the M2 tidal constituent drives tidal dynamics in the channel.

A common feature of past studies is that most of them focused on the shallow Zanzibar and Mafia channels [11,12]. As such very little is known of the ocean circulation in the Pemba Channel, which is deeper and rich in marine ecosystems including the pelagic fish resources and the coelacanth [3,13]. The pelagic fisheries are essential sources of food and sustain livelihoods of local communities [14]. The high catch of this fishery during the southeast monsoon season is associated with upwelling caused by strong winds that bring nutrient rich water into the productive layer [15]. Despite this importance, little is known of the surface circulation patterns along the Pemba Channel. Also, most of the available information had been derived from a few patchily distributed Eulerian current measurements [4,16] or model simulations [17]. The Pemba Channel is among the coastal areas with satellite-tracked drifter observations since June 1986, which provides an opportunity for filling the existing information gap of surface currents, the opportunity the present study explored.

The satellite-tracked drifters provide a robust tool for mapping changes in surface currents [18], as they provide measurements of the current speed and direction both in time and space [19]. Drifter’s observations have an advantage over conventional in-situ and ship-based measurements as they move with the water, tracking the near-surface current speed and direction [20] and pathways of water masses [21,22]. Several studies have used drifter observations to map and characterize the surface currents over the global ocean [8,18,19,22–24]. Similar studies have also been conducted in the Atlantic Ocean [20,25]. Drifters have also been used to explore the ocean circulation in the Indian Ocean. For instance [26] compiled 192 observations from drifters placed during 1975–1987 to map the surface current distribution in the tropical Indian Ocean. [27] studied Indian Ocean basin-scale surface currents using trajectories from satellite-tracked drifter observations, while [28] examined the surface circulation and seasonal variations in the Indian Ocean using drifter observations and Argo floats.

Although these studies revealed some important features of the general surface current circulation of the Indian Ocean, they are too coarse to resolve surface currents in the Pemba Channel. The presence of drifter observations in the Pemba Channel complements the missing long-term in-situ observations [17]. In this study, therefore, spatial and seasonal changes of surface current speed and direction in the Pemba Channel are examined using drifter observations.

## Materials and methods

### Study area

**Fig 1:**
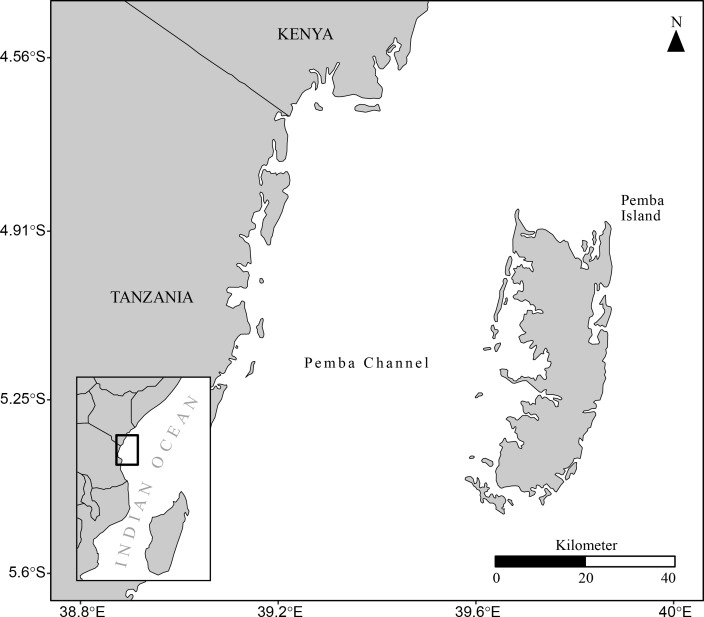
A map of the Pemba Channel. The inset map locates the Pemba channel (polygon with black color) in the Western Indian. The dataset used to draw these maps were obtained from the Institute of Marine sciences and **spData** package [30].

The Pemba Channel located between the Tanzania Mainland and Pemba Island (Fig 1) consists of a narrow coastal stretch, roughly 100 km long and 53 km wide with an average depth of about 300 m and a maximum depth of 800 m. Its geographic location is between longitude 39.0°E and 39.7°E and latitude 5.5 and 4.8°S (Fig 1). The weather and climate of the channel are both influenced by the monsoon winds [29], which reverse on annual basis to create two alternating seasons namely: southeast (SE) monsoon and northeast (NE) monsoon seasons [3]. The SE monsoon season, which occurs between May and September, is characterized by cooler temperatures, drier air conditions and the dominance of southerly winds of relatively high speeds. The NE monsoon, which occurs from November to March, is associated with warm weather conditions and northerly blowing winds of relatively low magnitude [3]. The periods during April and October, which are characterized by relatively calm weather, fall in the inter-monsoon season. The water flow in the channel is primarily driven by the East African Coastal current, which flows northward throughout the year.

### Data sources, processing and analysis

#### Basemaps and bathymetry

Several basemaps were used, including the country boundary shapefile and bathymetric data. The African continent’s countries boundary shapefile was obtained from the Institute of Marine Sciences (www.ims.udsm.ac.tz). The **sf** package for R software was used to read the ESRI^(R)^ shapefile and transform polygon’s country layer into simple features [31]. A one arc-minute global relief model of Earth’s surface that integrates land topography and ocean bathymetry was downloaded from the National Oceanic and Atmospheric Administration (NOAA) portal (https://www.ngdc.noaa.gov/mgg/global/). The dataset was imported into R’ workspace with the *read*.*asciigrid()* function of the **maptool** package [32] and removed the data outside the geographical extent of the Pemba Channel [33].

#### Drifter data

The drifter dataset from the Global Drifter Program was used to estimate surface currents of the Pemba Channel. The drifter observations within the Pemba Channel spanning from 1986 to September 2017 were downloaded from the Drifter Data Centre at the Atlantic Oceanographic and Meteorological Laboratory (AOML). The dataset includes geographical coordinates and subsequent eastern (U) and northern (V) [8, 22] velocities. These observations were processed and analyzed for each monsoon season—southeast (SE), northeast (NE) and inter–monsoon (IN) periods.

Processing the drifter data consisted of multi-step processes. First, the data file was imported into the workspace using the *read_table2()* function of **readr** package [35]. Second, the dataset was organized and consistently formatted into data frame to make easy plotting and analysis [33]. Formatting of the drifter dataset involved removal of some variables from the dataset that were not needed for analysis. An analytical markdown script code was created to iterate the process, and thus allowing viewing observations while processing and inspecting variables, and removing inaccurate observations from the dataset. The date variable was then decomposed into months and years variables [36], which were then used to sort the drifter observations according to their respective monsoon seasons—northeast (November to March), southeast (May to September) and inter monsoon (April and October) seasons [4,34].

Because the drifter observations in the Pemba Channel were sparsely distributed, it was important to use all the available data including drifters which had lost their drogues (sea anchors) and kept predominantly drifting under the influence of the surface winds. Such drifters gained an extra downwind motion because of direct wind forcing, Stokes drift, and other effects. To account for this phenomenon, 55% of undrogued drifters that had lost their drogues were corrected by interpolating ERA–Interim (https://www.ecmwf.int/en/forecasts/datasets/archive-datasets/reanalysis-datasets/era-interim) winds to the drifter locations. Then initial velocity fields were calculated from drogued drifters only and interpolated to the undrogued drifter locations. It was determined that an added downwind motion of 1.28% times the wind speed explained the difference between drogued and undrogued drifter velocities. The interpolated values from undrogued drifters matched well with those from drogued drifter as found in a recent study [22].

#### Sea surface height and wind speed

The data for the sea surface height (SSH) and QuikSCAT wind vectors were obtained from the Environmental Research Division’s Data Access Program (ERDDAP) server (https://coastwatch.pfeg.noaa.gov/erddap/index.html) using the **xtractomatic** package [37]. The ERDDAP server provides a simple and consistent way for downloading subsets of gridded and tabular scientific datasets in common file formats [37]. The date, longitude, and latitude variables from the drifters’ observations within the Pemba Channel were aligned to those of SSH and wind data. Once these variables were consistently matched, the daily wind speed and SSH data were then downloaded. For the Pemba Channel, the data for QuikSCAT and SSH spanned January 2000 to August 2009 and from March 1996 to December 2017, respectively. In both datasets, Only the grids and date of wind vector and SSH that matched the drifter date and location (longitude and latitude) were extracted.

#### Velocity calculation

Each drifter observation contains two orthogonal directions of zonal and meridional vectors, where the zonal vector is the current velocity toward the east and is referred to as the U component [38] and the meridional vector is the current velocity toward the north and is referred as the V component. The two components were used to calculate the speed of surface currents using the equation below:

$$V=\sqrt{U^{2}+V^{2}}$$

Where

*V* = Current velocity (ms^-1^);

*U* = Eastward velocity (ms^-1^);

*V* = Northward velocity (ms^-1^).

#### Drifter trajectories and surface current pathway

Each drifter observation possesses a unique Identification Number and position information (longitude, latitude), which were used to transform the drifter observations into trajectories using the **sf** package in R [31]. Each drifter produced one trajectory and the length of a trajectory depended on the number of drifter observations. The spheroid WGS84 datum was used as a coordinate reference system to place the drifter’s trajectories on the earth surface. Because the spheroid coordinates system units are in degrees, the degrees were transformed from the spheroid coordinate system of WGS84 to metric and planar coordinates system using Universal Transverse Mercator (UTM) zone 37 south projections. The transformed feature was then used for computing the distance between two drifter observations (points) and the total length of the drifter’s trajectory.

#### Gridding and surface current estimation

Before estimating the surface current speed, the drifter observations were separated depending on whether they fell within the SE or NE monsoon season. The filter function of **dplyr** package [33] was used to filter out observations outside the bounding box of the Pemba Channel. Because of the limited drifters’ observations, the NE and IN monsoon drifter observations were combined to into the NE season. Drifter observations were binned in 4 km grids and used to estimate the current speed within a bin for NE and SE seasons with Barnes algorithm [39].

#### Data analysis and mapping

For comparison analysis, the data were transformed and structured in tabular format where each cell contained an observation and each column contained a variable. The date variable of the observations was aligned with the NE, SE and IN seasons. Averaged monthly and seasonal surface currents, wind speeds and SSH were then computed and analyzed. The statistical parameters were calculated to compare the differences in wind speeds, current velocities and sea surface heights during monsoon seasons. A spearman test was used to test for significant associations between surface current velocities and winds and sea surface heights [40], while the Kruskal-Wallis test was used to test significant seasonal variations [41].

## Results

**Fig 2:**
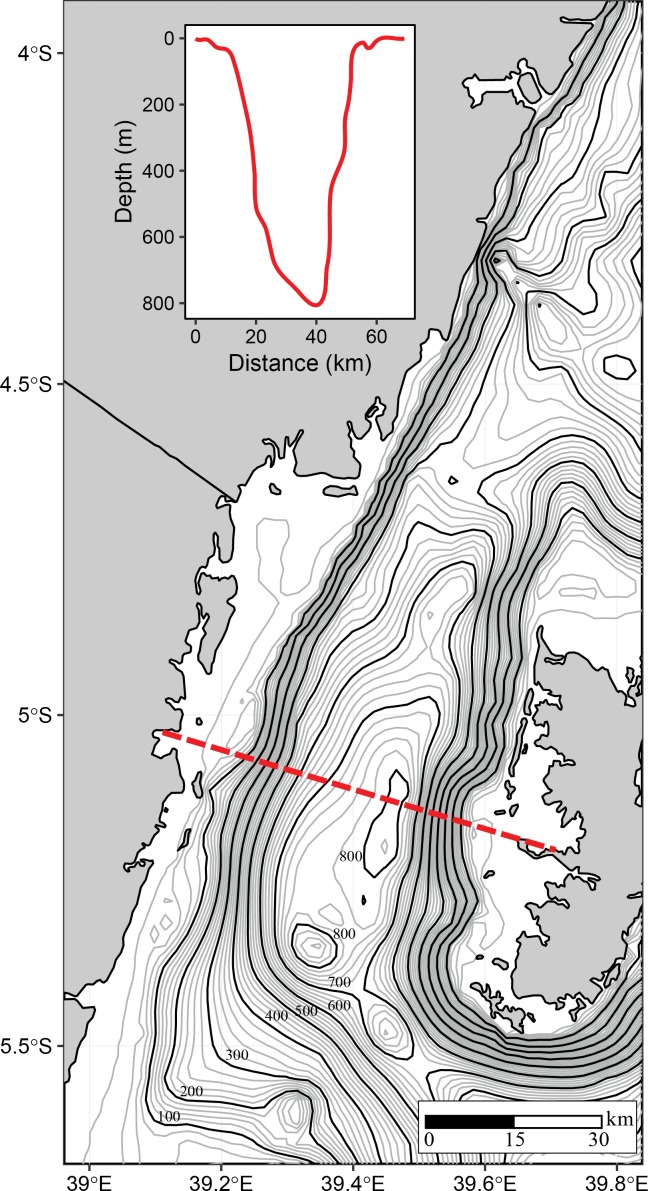
A map showing the sea bottom topography of the Pemba channel. Black isobars are hundred meters contour intervals and gray isobars are twenty meters contour interval. The inset plot shows the bathymetry bottom topography of the Pemba channel along an east-west transect indicated by a red dotted line.

Fig 2 shows the Pemba Channel bathymetry with contour lines representing the sea bottom topography. In the map of inset in Fig 2 a cross section of the sea bottom topography along, an east-west transect that is indicated by red dotted line is also presented. The Pemba Channel is characterized by a “central deep”, a feature which is about 800 m in depth near longitude 39.5°E and latitude 5.35°S. The sea bottom topography that is shallower than 100 m is characterized by a relatively gentle topographic gradient, where the bathymetry changes from 0 to 50 m within about 13 kilometers from the coastline. By contrast the sea bottom topography beyond 50 meters is characterized by a steeper topographic gradient, where the bathymetry changes from 50–600 m within 12 km (Fig 2).

**Fig 3:**
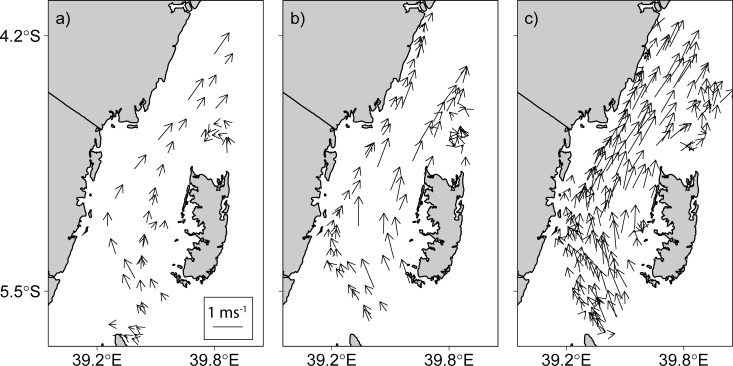
**Vector field velocity of current speed and direction in the Pemba Channel during a) northeast (NE), b) inter-monsoon (IN) and c) southeast (SE) monsoon seasons**.

The Global Drifter Program (GDP) dataset released in December 2017, has more than four million drifter observations in the tropical Indian Ocean region, bounded between longitude 15°S and 80°E and latitude 25°S and 15°N. Three-hundred-twenty-eight (328) six-hour interval observations collected by 45 unique drifters were found in the Pemba Channel. Only random samples of twelve observations are shown in Table 1. The SE monsoon had 197 drifters with velocities ranging from 0.04 to 1.73 ms^-1^ and a median speed of 0.84 ms^-1^. While the IN season had 80 drifters with velocities range from 0.06 to 1.57 and a median speed of 0.61 ms^-1^, 51 drifters crossed the Pemba Channel during the NE season and had velocities ranging from 0.04 to 1.34 ms^-1^ and a median velocity of 0.57 ms^-1^ (Table 2). Fig 3 shows the surface current speeds and direction in the Pemba Channel for the three seasons (SE, NE and IN monsoon) in the channel. The presented results provide a good coverage of drifter observation in the Channel at both spatial and seasonal scales (Fig 3). As can be seen from Fig 3, the direction of surface current during the NE (Fig 3A), IN (Fig 3B) and SE (Fig 3C) seasons are dominantly northward. However, some vectors during the NE monsoon season showed a southward direction (Fig 3A).

**Table 1:** A random sample of observations from twelve unique drifters that crossed the Pemba channel at different times.

		Drifter Time		
Drifter ID	Count	Entered Date	Left Date	Hours Spent
8603091	5	1986-06-29	1986-06-30	24
70973	6	2011-01-21	2011-01-22	24
64726990	6	2017-07-03	2017-07-04	24
114803	6	2013-06-01	2013-06-02	24
116212	11	2014-10-25	2014-10-27	48
90506	6	2010-05-22	2010-05-23	24
79023	5	2008-12-28	2008-12-29	24
147140	9	2017-03-27	2017-03-29	48
109404	4	2013-06-30	2013-07-01	24
109493	11	2014-09-22	2014-09-24	48

**Table 2:** Summary statistics of current velocities from drifter observations during different seasons in the Pemba Channel.

		Surface Velocity (m/s)				
Season	Count	Minimum	Maximum	Mean	Median	SD
SE	197	0.04	1.73	0.83	0.84	0.44
IN	80	0.06	1.57	0.67	0.61	0.38
NE	51	0.05	1.34	0.56	0.57	0.31

**Fig 4:**
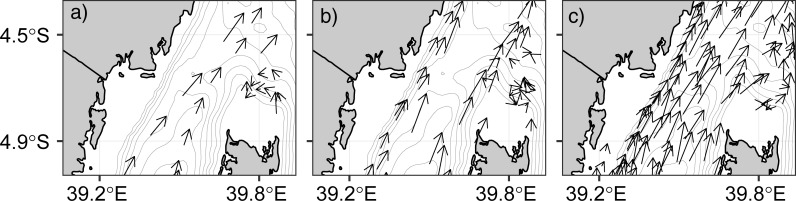
**Vector field showing cyclic motion of current north of the Pemba Island during a) NE, b) IN and c) SE seasons**. The gray isodepth represent hundred-meter interval contour lines.

Fig 4 shows swirling of surface currents north of the Pemba Island. During the NE monsoon (Fig 4A) and Inter-monsoon (Fig 4B) periods, currents tend to swirl at about longitude 39.8°E and latitude 4.7°S. The currents at this location move in a direction that is different from that of the main current. Rather than moving north, they swirl and create a circular motion. However, the distinctive swirling feature found during the NE and IN period is unclear during the SE season (Fig 4C). The directionality of the swirling appears to be constrained to follow the bathymetry during the NE and IN periods. This result suggests that under those wind regimes, the drifters may be following potential vorticity contours north of Pemba Island while this constraint is relaxed during the SE monsoon [9]. The cyclic motion of current vectors suggests the presence of localized eddies north of the Pemba Channel.

**Fig 5:**
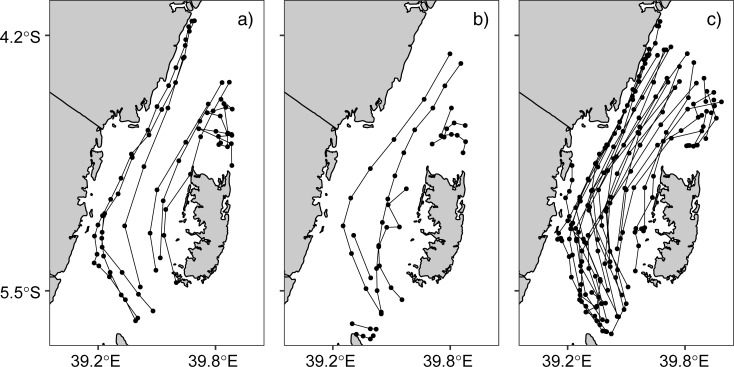
**Drifters’ Trajectories in the Pemba Channel during a) IN, b) NE, and c) SE monsoon seasons**.

Fig 5 shows the drifter trajectories in the Pemba Channel during the SE, NE, and IN seasons. The presented results show highest density (i.e 28 trajectories) in the channel during the SE season (Fig 5C), compared to nine and eight trajectories during the IN (Fig 4A) and the NE season (Fig 5B), respectively.

**Fig 6:**
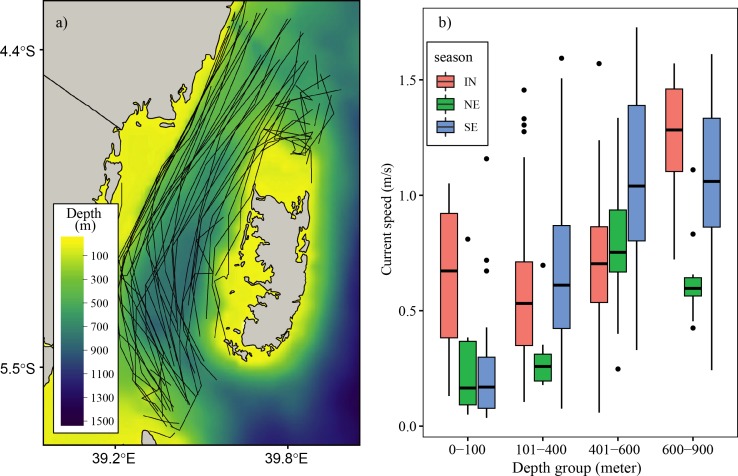
**A map of the Pemba Channel showing a) the relationship between the drifter trajectories and bathymetry and b) boxplot showing the surface current velocity at different depth intervals.** The black dots in the boxplot indicate outliers.

Most trajectories of the drifters in the Pemba Channel were found to drift in waters deeper than 100 meters (Fig 6A). Out of the 328 drifters in the channel, only 36 drifters (10 percent) were found in waters below 100 meters while the remaining 209 drifters (90 percent) were found drifting in waters deeper than 100 meters (Table 3). In general, the surface current velocities in the Pemba Channel vary seasonally and with water depth. The weaker current velocities were found in waters below 100 meters while strong current velocities were found in deep waters (Fig 6B and Table 3)

**Table 3:** Summary statistics of drifters at different water depth intervals.

		Surface velocity (m/s)		
Depth	Count	Median	Mean	SD
0–100	36	0.2	0.32	0.31
101–400	141	0.55	0.61	0.36
401–600	85	0.93	0.96	0.38
600–900	66	0.99	0.98	0.35

**Fig 7:**
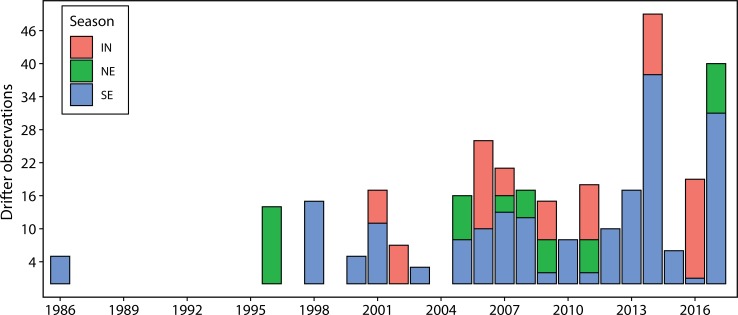
The annual number of drifter observations in the Pemba Channel grouped by seasons.

Fig 7 shows the number of drifter observations in the Pemba Channel from 1986 to 2017. Prior to 2005, the total number of drifter observations in the channel varied from year to year, with considerable data gap in some years. However, continuous drifter observations in the channel started in 2005. The results show that the drifter observation since 2005 varied from 6 to 48 per year with an averages of 18 drifter observations per year. The SE monsoon has higher drifter observations compared to the NE and IN seasons. Furthermore, the year 2007,2009 and 2011 had drifter observations that covers the NE, SE and IN season, while the remaining years had drifter observation covering either one or two seasons (2005, 2010), IN and SE monsoon seasons (2006, 2010), and IN and NE monsoon seasons (2011). The SE monsoon season has been sampled with drifter observations every year except in 2011 and since 2012 onwards, drifter arrays have been passing through the Pemba Channel during the SE monsoon season.

**Fig 8:**
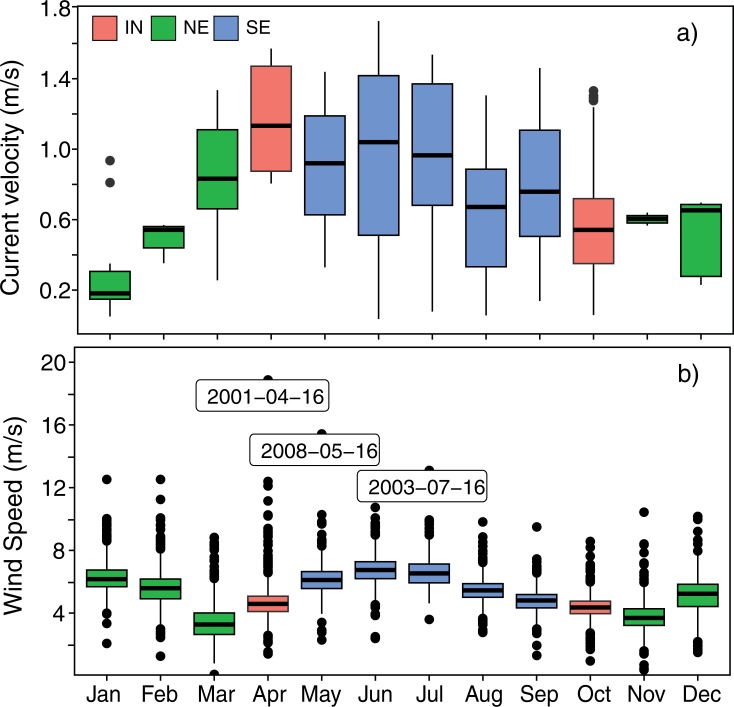
**Boxplot showing monthly variation of a) surface current velocity and b) wind speed in the Pemba Channel**. The dates show days with anomalous highest wind speeds.

Fig 8 shows the monthly variation of surface current speed and wind speed within the Pemba Channel. The monthly median current speed ranged from 0.183 ms^-1^ in January to 1.13 ms^-1^ in April with an average speed of 0.74 ms^-1^ (Table 4). Furthermore, the results show that highest variation of surface current speed is experienced during March-October and December, and the lowest variation of surface current speeds is experienced in November, January and February (Fig 8A). The SE period experience high current velocity (mean ±SD = 0.832 ± 0.43, median = 0.837), which was followed by the IN season (mean ± SD = 0.67 ± 0.38; median = 0.61 ms^-1^), while the lowest current velocities (mean ± SD = 0.56 ± 0.31; median = 0.57 ms^-1^) occurred during the NE monsoon season (Table 5).

**Table 4:** Monthly Statistical metrics of surface current speed from the drifters in the Pemba Channel.

		Surface Current Speed (m/s)				
Month	Count	Min	Max	mean	Sd	median
Jan	12	0.05	0.94	0.30	0.30	0.18
Feb	8	0.35	0.57	0.49	0.08	0.54
Mar	14	0.26	1.34	0.82	0.35	0.83
Apr	12	0.80	1.57	1.17	0.30	1.13
May	34	0.33	1.44	0.92	0.32	0.92
Jun	46	0.04	1.73	0.93	0.53	1.04
Jul	37	0.08	1.54	0.92	0.47	0.97
Aug	49	0.06	1.31	0.64	0.34	0.67
Sep	31	0.14	1.46	0.79	0.40	0.76
Oct	68	0.06	1.33	0.58	0.32	0.54
Nov	7	0.57	0.64	0.60	0.03	0.60
Dec	10	0.23	0.70	0.50	0.21	0.65

**Table 5:** Seasonal Statistical metrics of Surface current speeds in the Pemba Channel during the southeast (SE), northeast (NE) and inter-monsoon (IN) seasons.

		Surface Current Speed (m/s)				
Season	Count	Min	Max	Mean	Sd	Median
IN	80	0.06	1.57	0.67	0.38	0.61
NE	51	0.05	1.34	0.56	0.31	0.57
SE	197	0.04	1.73	0.83	0.44	0.84

The difference in mean surface current between NE, IN and SE monsoon season was significant (F_(2,245)_ = 40.74, *p* < 0.05), with higher mean surface current velocity during the SE compared to the IN (*p* = 0.009) and NE monsoon (*p* < 0.05). Although the mean current velocity during the IN periods was higher than the NE monsoon (Table 5), the difference was insignificant (post-hoc test, *p* = 0.283). The monthly wind speed variations in the Pemba Channel are shown in Fig 8B. The channel has a median wind speed of 5.34 ms^-1^, which varied from 3.27 ms^-1^ in March to 6.75 ms^-1^ in June. The strong winds blow in the channel from May through August and weaken in September. The channel experiences winds with speeds below 4 ms^-1^ during March and November. The highest wind speed of about 18 ms^-1^ was observed in April.

**Fig 9:**
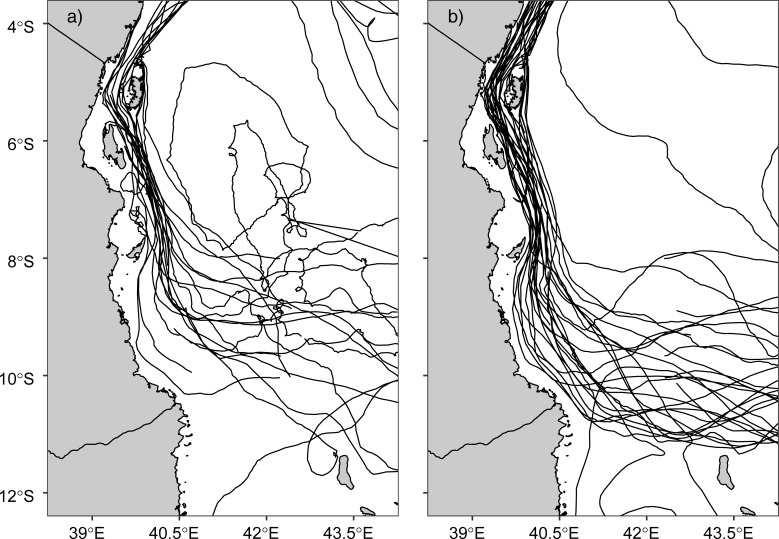
**Trajectories of drifters that passed through the Pemba channel during a) the NE and b) SE monsoon season**.

Fig 9 shows trajectories of drifters originating from the tropical Indian Ocean and eventually entered the Pemba Channel during the SE (Fig 9B) and NE (Fig 9A) seasons. On reaching the southern Tanzania coastal waters (near latitude 11°S and longitude 41°E), the drifter trajectories follow the northward flowing East African Coastal Current (EACC), It is worth noting that no drifter trajectories along the Tanzania waters passed through the Mafia and Zanzibar channels and instead all the drifter trajectories passed east of the two islands. On reaching the northern parts of Zanzibar Island nearly latitude 6°S, the drifter trajectory split into two sets: one set drifting between the Pemba and Zanzibar islands and then northward along the Pemba Channel and the other set drifting along the eastern side of the Pemba Channel. The two trajectories eventually converged on the northern parts of the Pemba Channel and drifted further northward towards the Kenyan coastal waters. Furthermore, the northward drifting of the drifters were observed during both the SE and NE monsoon seasons (Fig 9).

**Fig 10:**
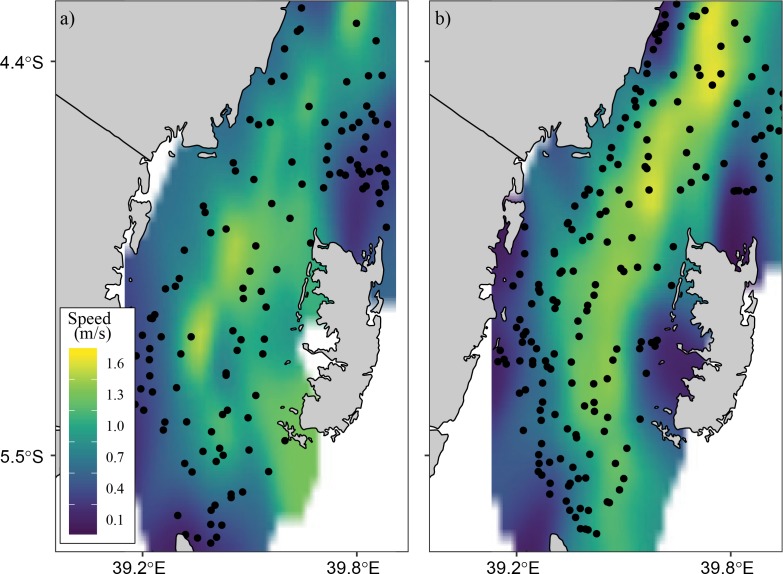
**Spatial pattern of surface current velocities in the Pemba Channel during a) NE and b) SE monsoon seasons**. Drifter observations are overlaid as circular points.

Fig 10 shows the spatial pattern of surface current speeds in the Pemba Channel during the NE and SE monsoon seasons. The results show that highest current speeds exceeding 1.3 ms^-1^ are generally observed along the central parts of the Channel and lower current speeds (less than 1.3 ms^-1^ are found on the shallow waters along the eastern and western parts of the Channel. Furthermore, during the NE monsoon season the highest current speeds (>1.3 ms^-1^) occurs as few patches on the central parts of the Channel (Fig 10A), while during the SE monsoon season the highest current speeds (>1.3 ms^-1^) occur as a continuous feature along the entire central parts of the Channel (Fig 10B).

### Seasonal variation of wind speed

**Fig 11:**
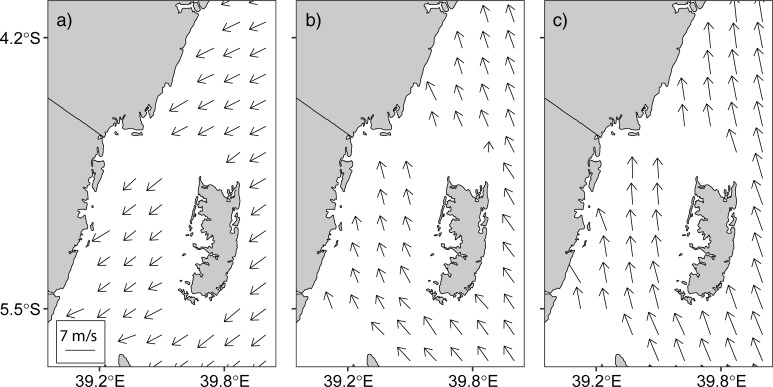
**Wind vectors derived from QuikSCAT showing wind speed and direction in the Pemba Channel during a) NE, b) IN and c) SE monsoon seasons**.

The spatial distribution of wind vectors along the Pemba Channel as deduced from the QuikSCAT data are presented in Fig 11 and S1 Fig. The results show that the winds are generally northerly (blowing from north to south) during the NE monsoon season (Fig 11A) and southerly (blowing from south to north) during the IN and SE monsoon seasons (Fig 11B & 11C). Analyses of the data using Kruskal-Wallis test revealed that the wind speeds along the Pemba channel varied with season, with lowest wind speeds during the IN period (Mean ± SD = 4.57 ± 1.16) compared to the NE monsoon season (Mean ± SD = 4.86 ± 1.56). Highest wind speeds were observed during the SE monsoon season (Mean ± SD = 5.95 ± 1.13) (Kruskal-Wallis, W_(2)_ = 1390.8, *p* < 0.001).

**Fig 12:**
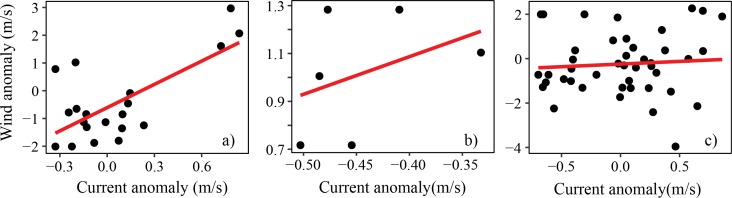
**The relationship between wind speed anomaly and current speed anomaly in the Pemba Channel during a) IN, b) NE and c) SE monsoon seasons**.

**Fig 13:**
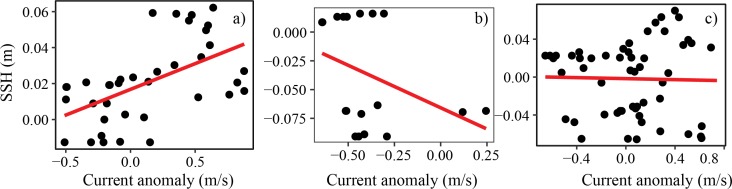
**The relationship between Sea Surface Height (SSH) anomaly and surface current velocity anomaly in the Pemba Channel during a) IN, b) NE and c) SE monsoon seasons**.

Fig 12 compares the relationship between wind speed and current velocity in the Pemba Channel during the different seasons. The NE (Fig 12A) and IN (Fig 12B) seasons are characterized by positive relationships, suggesting that strong winds favor the development of strong current velocities. No apparent relationship was observed for the SE monsoon season. Fig 13 compares the relationship between current speed anomaly and sea surface height anomaly on the Pemba Channel. While the NE monsoon season is characterized by positive relationship (Fig 13A), the IN is characterized by a negative association (Fig 13B), and no apparent relationship was evident during the SE season (Fig 13C).

**Fig 14:**
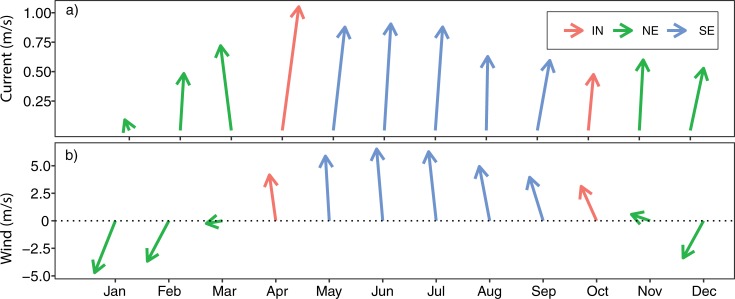
**Climatological median of a) surface current speed from drifter observations and b) wind speed from QuikSCAT in the Pemba Channel**.

The eastward current velocity (U) and northward current velocity (V) from the drifter observations were used to deduce the monthly and seasonal current vectors (speed and direction) for surface currents (Fig 14A) and winds (Fig 14B) in the Pemba Channel. The results presented in Fig 14A shows that the current in the Pemba Channel flows northward throughout the year at varied speed, while the winds strength varies both in speed and direction with seasons. The wind direction is southerly (blowing from the south) during the SE and IN and reverses to northerly during the NE monsoon season (Fig 14B)

## Discussion

The present study is the first to deduce the surface current patterns using drifter observations in Tanzanian waters. It is also the first case study that provides detail of the nature of surface current circulation in the Pemba Channel, where ship-based oceanographic research work is constrained by harsh sea conditions. The Pemba Channel is the least explored channel compared with the Mafia and Zanzibar channels. Previous ocean circulation studies along the coastal waters of Tanzania focused on the shallow channels of Zanzibar and Mafia and the dynamics of the East African Coastal Current (EACC) [17,10,9,4]. Lack of hydrographic data hindered studies on the structure and variability of currents in the Pemba Channel. The available information on the ocean circulation in this channel came from sparsely-located measurements [9] and numerical modeling [17]. Contrary to previous work, this study used drifter observations to understand the surface circulation in the channel. A sample of 328 drifter observations recorded at different monsoon seasons provided a broad picture of surface current dynamics in the channel. Besides, this study demonstrate that areas with limited in-situ ship-based data but with drifter observations can be investigated using the approaches that have been used in this study.

Although the presented drifter observations had considerable data gaps prior to 2005 (Fig 7), the data spanning from 2005 to present was good enough to permit us to determine the monthly-mean of surface current speeds (Fig 8) and the seasonal pattern of the surface current speeds (Table 5) of the investigated area. The study revealed that the SE season was characterized by higher median surface current speed of about 0.84 ms^-1^ compared to 0.57 ms^-1^ of the northeast (NE) season. These findings are consistent with other previous studies qualitatively [4,10,17], which reported stronger current speeds during the SE monsoon season compared to the NE monsoon season. However, the current speeds reported here are slightly lower. For example, [4] found current velocity of 2.6 ms^-1^ during the SE and 1.60 ms^-1^ during the NE seasons in the Zanzibar Channel, which is three and two folds higher than our results. However, our study was conducted in the Pemba Channel while most previous studies are from the Mafia and Zanzibar channels. Therefore, the difference in the study areas and mismatch of the analyzed data are possible reason for the noted difference in current speeds.

The marked seasonal variation of the surface current along the Pemba Channel is attributed to the monsoon winds [3,42], which also vary with seasons (Fig 11). The SE season, which span from early May to September is characterized by strong wind which blows from the south [16] and would tend to drag the surface waters toward the north. With the wind reversal during the NE monsoon season (November to March), the surface waters would tend to drag the surface waters toward the south. However, since the winds are relatively weaker, the EACC is not reversed during the NE season. As a result, the surface current during the NE season continue to flow northward against the wind direction (Fig 14) The wind speed vectors in the Pemba Channel during the NE (Fig 11A) and SE (Fig 11C) seasons agree quite well in direction with the monsoon seasons in the Tropical Indian Ocean region.

Comparing the current speeds (Fig 14A) with wind speeds (Fig 14B), the study found that currents and winds in the Pemba Channel flow in the same direction during the SE and IN seasons, but the association was unclear (Fig 12B & Fig 12C). Similarly, the sea surface height showed an unclear relationship with surface current (Fig 13C). In contrast, during the NE season, winds reverse and flow in opposite direction to current flow (Fig 14) with strong positive association (R^2^ = 0.69, Fig 12A)

The stronger southeasterly winds during the SE monsoon cause the surface current to have higher speeds (Fig 3C) compared to the NE season (Fig 3A). The weak current speed during the NE is contributed by reversing winds (Fig 11A), which blow against the current (Fig 3A & Fig 14B). These findings suggest that apart from the winds, there are other factors determining current speed in the channel. For example, the EACC plays an important role for the circulation in the Pemba Channel. Although the seasonal variability in wind speed (Fig 11) lead to seasonal variation of northward flow current speed (Fig 3), the reversing wind direction during the NE season (Fig 11 & Fig 14) does not reverse the direction of the current flow. This shows how important the EACC is to the observed ocean circulation, which pumps its water through the channel throughout the year (Fig 9) [44]. The EACC is the strongest western boundary current that flows northward along the East African coast [44]. At latitude 4°S, the EACC splits and part of it turns northwest and enters the Pemba Channel (Fig 9). Previous studies show clearly that most of the westward transport toward the EACC occurs south of the Tanzanian waters at 9°S. Similar to [44], this study found the westward flow feeding into the EACC occurs between latitudes ~9°S and ~11°S (Fig 9). The EACC pathway is much clearer during the SE (Fig 9B) than NE monsoon season (Fig 9A)

Weaker current flow of the EACC during the NE monsoon season affects the current speed in the Pemba Channel (Fig 10A). The strong EACC and the reversing Somali Current (SC) during the SE season creates a continuous northward current that strengthens the current in the Pemba Channel (Fig 10B). The way the EACC vary also affects the spatial variation of the current speed in the channel. For instance, the median surface current speed of above 1.20 ms^-1^ flowing northward is confined in the area between ~39.4°E and 39.7°E (Fig 10B), which has a water depth greater than 200 meters (Fig 2). During the SE (Fig 10B), currents below 0.8 ms^-1^ are found along the shallow areas of the channel (Fig 2). By contrast, the spatial pattern of the estimated surface current during the NE season showed no clear pattern but was characterized by patches of strong and weak surface current speeds along the channel (Fig 10A). The observed patches of currents during the NE season could have resulted from poor drifter observations (Fig 3A & 3B). Also, the unusually strong wind speeds in April (Fig 8B) may have increased the current speeds (Table 4).

This study found that most drifters that passed through the Pemba Channel drifted with water from the North Equatorial Madagascar Current (NEMC) that propagates westward (Fig 9, [43]). The results further explained connectivity pathways between these currents and the South Equatorial and East African Coastal Currents. Nonetheless, the results were consistent with the assumption that the NEMC splits into northern and southern flows at latitude 11°S, thus forming the East African Coastal Current [43,44]. While the drifters’ observations clearly revealed that part of the northward flow of the EACC splits and enters the Pemba Channel during the SE (Fig 9B) and NE monsoon season (Fig 9A), the study found some drifters flew in opposite direction to the EACC during the NE monsoon (Fig 3B). This observation suggests that the EACC does not always flow northwards but rather reverses its direction with the prevailing winds. [4] reported similar findings in the Zanzibar Channel, and stated that this is a common phenomenon along the Tanzanian coastal waters.

One fascinating finding of this study is the swirling surface current pattern north of the Pemba Channel (Fig 4). Near ~39.8°E and ~4.7°S, the current flows in a cyclic direction during the NE (Fig 4A) and IN seasons (Fig 4B), a pattern that disappears during the SE period (Fig 4C). Similar monsoon induced cyclic motion (eddies) along the Pemba Channel have been reported by [46]. These cyclic motions are considered to enhance vertical transport of nutrients into surface waters around the Pemba Island. The weakening of the sea surface height (Fig 13) and the reversed winds during the NE season (Fig 11A), and conservation of potential vorticity partly contribute to the cyclic motion [45]. The formation of the Equatorial Counter Current (ECC) during the NE period when the EACC meets the southward flowing Somali Current near 3°S may also explain the observed cyclic flow of surface current north of the Pemba Island (Fig 4).

Finally, the use of drifter observations in this study has improved our understanding of the way surface current vary in space and time in the Pemba Channel. More importantly, the presented results show the spatial variation of surface current speed and direction in the Pemba Channel and how they vary with seasons (see Fig 10). The small numbers of drifter observations (Fig 5) explain the unclear flow patterns during the NE and IN seasons. Nonetheless, the circulation patterns are important oceanographic features that need further exploration, especially using the drifter data which offers a more robust avenue to the solution. But the study provides clear evidence that the EACC reverses with seasons in the shallow parts of the Tanzanian coastal waters (Fig 3). These results highlight the need for continued future placements of drifters in the region. More drifter observations are required to further study the seasonal and inter-annual variability in surface circulations in the Pemba Channel.

## Conclusion

Information of surface current variations in the Pemba Channel and many other coastal areas in the Western Indian Ocean is limited because of lack of consistent and time-series of oceanographic data. The bottom topography of the Pemba Channel partly hinders installation of equipment needed to collect continuous and high-frequency data. However, since the late 1980s the Pemba Channel has been traversed by drifters from the Global Drifter Program, which offers long-term in-situ observations to study surface currents [22]. Despite the available drifter observations, the Pemba Channel is the least explored coastal waters of Tanzania [17]. This study used drifter observations to understand how surface current speeds vary in time and space in the Pemba Channel. We have also deduced that the EACC does not always flow northwards, but changes its direction based on the monsoon seasons. Therefore, this study provides very important surface current information in the Pemba Channel. And although the drifters have proved to be robust tools for oceanographic data collection, even in areas without oceanographic observations [23], more drifter observations in the shallower parts of the Pemba Channel are required to expand this analysis.

## Supporting information

S1 Table The long formatted comma separated bathymetry file covering the Pemba Channel. The file contains three variables—longitude, latitude and depth.
(CSV)

S2 Table The long formatted comma separated file containing drifter observations and sea surface height (ssh) data. The variable date, longitude, latitude and season are common in both in drifter and sea surface height dataset. The U, V, SST and velocity variables found only in drifter dataset and the ssh is found in sea surface height dataset.
(CSV)

S3 Table The long formatted comma separated file containing drifter observations and QuikSCAT daily wind data. The variable date, longitude, latitude and season are common in both drifter and sea QuikSCAT. The U, V, SST and velocity are variables found in drifter alone and the x_wind, y_wind and wind speed are variable found only in QuikSCAT.
(CSV)

S4 Table A comma separate file containing drifter data. The dataset has eleven variables (id, longitude, latitude, drogue, u, v, SST, year, month, day and hour).
(TXT)

S1 Fig Animated monthly mean climatology wind speed and direction in the Pemba Channel. (GIF)
