# Supplementary figures and images for: Seasonal and spatial variation of surface current in the Pemba Channel, Tanzania
Source: PLoS One. 2019 Jan 7;14(1):e0210303. doi: 10.1371/journal.pone.0210303 (PMC6322782; doi:10.1371/journal.pone.0210303)

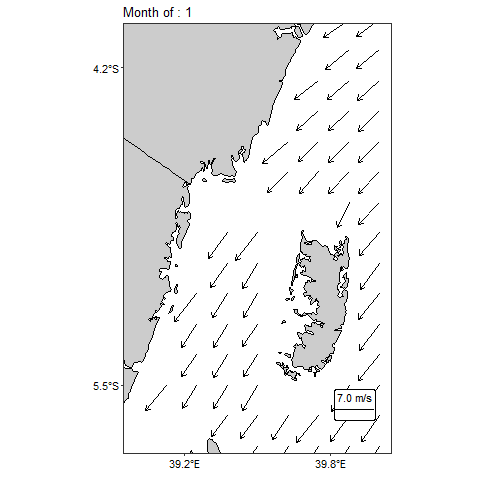

Supplement: S1 Fig — (GIF) [file pone.0210303.s005.gif]
